# Pleiotropic Effects of Myocardial MMP-9 Inhibition to Prevent Ventricular Arrhythmia

**DOI:** 10.1038/srep38894

**Published:** 2016-12-14

**Authors:** Ching-Hui Weng, Fa-Po Chung, Yao-Chang Chen, Shien-Fong Lin, Po-Hsun Huang, Terry B. J. Kuo, Wei-Hsuan Hsu, Wen-Cheng Su, Yen-Ling Sung, Yenn-Jiang Lin, Shih-Lin Chang, Li-Wei Lo, Hung-I Yeh, Yi-Jen Chen, Yi-Ren Hong, Shih-Ann Chen, Yu-Feng Hu

**Affiliations:** 1 Division of Cardiology, Department of Medicine, Taipei Veterans General Hospital, Taipei, Taiwan; 2 Faculty of Medicine, School of Medicine, National Yang-Ming University, Taipei, Taiwan; 3 Department of Biomedical Engineering, National Defense Medical Center, Taipei, Taiwan; 4 Institute of Biomedical Engineering, National Chiao-Tung University, Hsinchu, Taiwan; 5 Institute of Brain Science, National Yang Ming University, Taipei, Taiwan; 6 Division of Cardiology, Department of Internal Medicine, Mackay Memorial Hospital, Mackay Medical College, Taipei, Taiwan; 7 Division of Cardiovascular Medicine, Department of Internal Medicine, Wan Fang Hospital, Taipei Medical University, Taipei Taiwan; 8 Faculty of Medicine, Department of Biochemistry, Kaohsiung Medical University, Kaohsiung, Taiwan

## Abstract

Observational studies have established a strong association between matrix metalloproteinase-9 (MMP-9) and ventricular arrhythmia. However, whether MMP-9 has a causal link to ventricular arrhythmia, as well as the underlying mechanism, remains unclear. Here, we investigated the mechanistic involvement of myocardial MMP-9 in the pathophysiology of ventricular arrhythmia. Increased levels of myocardial MMP-9 are linked to ventricular arrhythmia attacks after angiotensin II (Ang II) treatment. MMP-9-deficient mice were protected from ventricular arrhythmia. Increased expressions of protein kinase A (PKA) and ryanodine receptor phosphorylation at serine 2808 (pS2808) were correlated with inducible ventricular arrhythmia. MMP-9 deficiency consistently prevented PKA and pS2808 increases after Ang II treatment and reduced ventricular arrhythmia. Calcium dynamics were examined via confocal imaging in isolated murine cardiomyocytes. MMP-9 inhibition prevents calcium leakage from the sarcoplasmic reticulum and reduces arrhythmia-like irregular calcium transients via protein kinase A and ryanodine receptor phosphorylation. Human induced pluripotent stem cell-derived cardiomyocytes similarly show that MMP-9 inhibition prevents abnormal calcium leakage. Myocardial MMP-9 inhibition prevents ventricular arrhythmia through pleiotropic effects, including the modulation of calcium homeostasis and reduced calcium leakage.

Ventricular arrhythmia is the leading cause of mortality commonly linked to a structurally abnormal heart^1^,^2^. Recent advances in implantable cardioverter-defibrillator therapy and catheter ablation have enabled significant progress but remain associated with surgical complications and electronic malfunctions^3^,^4^. A limited number of pharmacological treatments, primarily antiarrhythmic drugs, are associated with complications that include lung fibrosis, hyperthyroidism, pro-arrhythmias, and cardiac dysfunction^5^. The underlying pathophysiological mechanisms of ventricular arrhythmia remain incompletely understood, despite the identification of alterations in intracellular calcium handling, electrical remodeling, intercellular uncoupling (Connexin 43, Cx43), and fibrosis as major contributors to arrhythmia^6^,^7^. Recent work has further highlighted the importance of leaky channels, including ryanodine receptors (RyR2). The open probability of RyR2 increases after hyperphosphorylation, thus leading to Ca^2+^ leakage from the sarcoplasmic reticulum (SR), which depolarizes cardiomyocytes and triggers fatal arrhythmia^6^,^8^.

Matrix metalloproteinase-9 (MMP-9) is a zinc-dependent endopeptidase that regulates pathological cardiac remodeling processes that are involved in fibrosis and inflammation^9^,^10^,^11^. MMP-9 directly degrades extracellular matrix (ECM) proteins and activates cytokines to regulate tissue remodeling^9^,^10^,^11^. Cardiomyocytes are an active reservoir of MMP-9^12^. However, the functional role of MMP-9 in cardiomyocytes is not well understood, despite having been studied in a variety of cell types^9^,^10^,^11^,^12^. Clinical studies have suggested that myocardial MMP-9 is increased in patients with cardiac dysfunction^13^,^14^,^15^, and high serum levels of MMP-9 are associated with increased ventricular arrhythmia and sudden cardiac death^16^,^17^,^18^; however, whether the relationship between MMP-9 and ventricular arrhythmia is causal or an epiphenomenon is not clear. Furthermore, the mechanisms linking MMP-9 and ventricular arrhythmia have not been clearly described. One possible mechanistic link by which MMP-9 might contribute to ventricular arrhythmia is cardiac fibrosis and intercellular uncoupling, whereas cardiomyocyte-specific mechanisms, such as calcium dysregulation, might represent an alternative hypothesis. Here, we use translational approaches in animal models and human induced pluripotent stem cell-derived cardiomyocytes (hiPSC-CMs) to demonstrate the causal link between MMP-9 and ventricular arrhythmia, study the mechanisms underlying MMP-9 inhibition, and explore its translational potential.

## Results

### MMP-9 deficiency prevents ventricular arrhythmia in a mouse model

**Figure 1:**
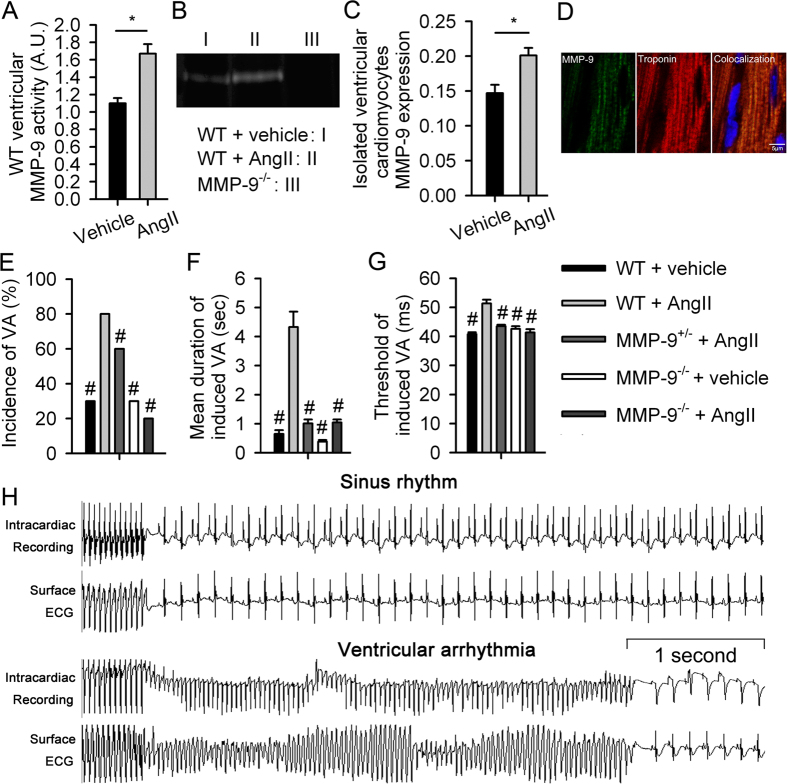
MMP-9 deficiency prevents ventricular arrhythmia. (**A**) MMP-9 enzymatic activity in the ventricular tissue after Ang II treatment in WT mice was assessed by zymography (n = 4, *P < 0.05). (**B**) Representative image of MMP-9 ventricular activity by zymography. Cropped blots are displayed, and full-length blots are included in Supplementary Fig. S10. (**C**) MMP-9 protein expression in the isolated ventricular cardiomyocytes as determined by Western blot analysis (n = 4, *P < 0.05). (**D**) Colocalization of MMP-9 and myofibrils. (**E** to **H**) The incidence (**E**) and duration (**F**) of ventricular arrhythmia episodes after the burst ventricular stimulation and ventricular arrhythmia induction threshold (**G**) were partially reduced in the MMP-9^+/−^ mice and completely prevented in the MMP-9^−/−^ mice (n = 10 for each group, ^#^*P* < 0.05 *vs.* WT + Ang II). (**H**) Representative ECG and intracardiac tracing after the burst ventricular stimulation. Polymorphic ventricular tachycardia was induced in the WT mice after Ang II treatment. However, ventricular arrhythmia was not inducible in the MMP-9^−/−^ mice after Ang II treatment. Data are shown as the mean ± SEM.

All of the characteristics of the mice are listed in Supplementary Tables S1 through S3. There were no differences in electrophysiological (EP) characteristics and ventricular function between MMP-9 homozygous knock-out mice (MMP-9^−/−^) and wild-type (WT) littermate mice. MMP-9 activity increased in the ventricular tissue following angiotensin (Ang II) treatment (Fig. 1A and B), as confirmed by protein expression (0.64 ± 0.10 vs. 0.27 ± 0.02, *P* = 0.01, n = 6 by Western blot). MMP-9 is secreted by a variety of cells, including fibroblasts and immune cells^10^,^11^. Therefore, we attempted to characterize MMP-9 protein expression in isolated ventricular cardiomyocytes, which express higher levels of MMP-9 after Ang II treatment in WT mice (Fig. 1C). Immunofluorescence staining revealed clear myocardial expression of MMP-9, which colocalized with myofibrils (Fig. 1D). Upon epicardial ventricular EP stimulation, the WT mice challenged with Ang II showed a higher susceptibility to ventricular arrhythmia than vehicle-treated WT mice and presented a higher incidence and duration of ventricular arrhythmia episodes, as well as a lower threshold to ventricular arrhythmia induction (Fig. 1E–H). The longest ventricular burst pacing interval that induced ventricular arrhythmia was defined as the induction threshold. Longer pacing intervals required to induce ventricular arrhythmia were correlated with a greater ease of ventricular arrhythmia induction, accounting for 13.6% of monomorphic ventricular tachycardias and 86.4% of polymorphic ventricular tachycardia/fibrillation. Ang II-induced ventricular arrhythmia vulnerability was completely prevented in the MMP-9^−/−^ mice. Furthermore, a dose-dependent reduction in ventricular arrhythmia was observed between the WT, MMP-9^+/−^ and MMP-9^−/−^ mice. This result suggests that MMP-9 is mechanistically involved in ventricular arrhythmia induction. MMP-2 activity was not associated with the reduction of ventricular arrhythmia (Supplementary Fig. S1).

### MMP-9 deficiency partially reverses ventricular fibrosis and Cx43 distribution

**Figure 2:**
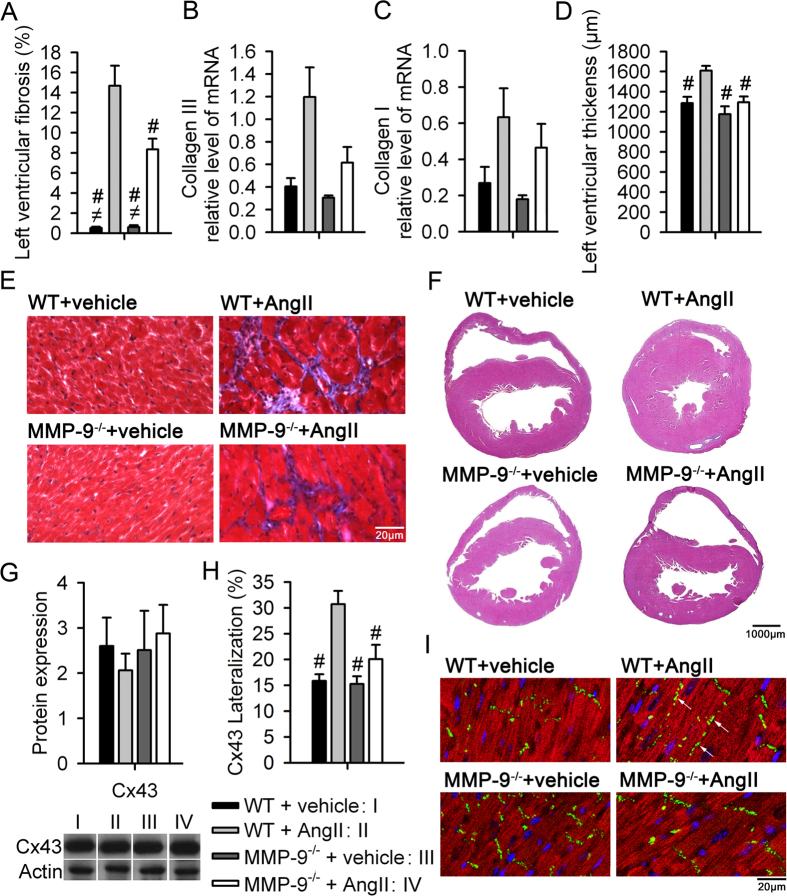
MMP-9 deficiency partially decreases cardiac fibrosis. (**A**) Quantitative analysis of cardiac fibrosis (n = 4–5, ^#^*P* < 0.05 *vs.* WT + Ang II, ^≠^*P* < 0.05 *vs.* MMP-9^−/−^ + Ang II). (**B** and **C**) The collagen III and collagen I mRNA levels did not differ between WT and MMP-9^−/−^ mice after Ang II treatment (n = 4–6). (**D**) Left ventricular thickness was increased in WT mice but not in MMP-9^−/−^ mice (n = 6–12, ^#^*P* < 0.05 *vs.* WT + Ang II). (**E**) Representative image of Masson’s trichrome stain used to delineate cardiac fibrosis. (**F**) Representative image of cardiac hypertrophy illustrated by H&E staining. (**G**) Quantitative analysis of Cx43 protein expression and representative image of a Western blot. Cropped blots are displayed, and full-length blots are included in the Supplementary Fig. S11. (**H**) Cx43 lateralization was prevented in MMP-9^−/−^ mice after Ang II treatment (n = 8–11, ^#^*P* < 0.05 *vs.* WT + Ang II). (**I**) Representative image of Cx43 lateralization (denoted by white arrows; Green: Cx43, red: troponin T, blue: DAPI). Data are shown as the mean ± SEM.

Cardiac fibrosis (Fig. 2A) increased in WT mice after Ang II treatment (*P* < 0.001), which was partially reversed in the MMP-9^−/−^ mice. However, Collagen I and III mRNA expression confirmed no reduction of collagen in the MMP-9^−/−^ mice compared to the WT mice after Ang II treatment (Fig. 2B and C). Ang II treatment in the WT mice led to left ventricular hypertrophy, which was prevented in the MMP-9^−/−^ mice (Fig. 2D). Representative images are shown in Fig. 2E and F. Differences in the expression of Cx43 were not observed between the WT and MMP-9^−/−^ mice (Fig. 2G)^19^. However, the lateralization of Cx43 increased in the WT mice after Ang II treatment, which was prevented in MMP-9^−/−^ mice (Fig. 2H and I). MMP-9 deficiency did not reduce Ang II-induced cardiac inflammation and oxidative stress (Supplementary Fig. S2). The optical mapping was used to record action potentials and conduction velocity in mice ventricles. The AngII treatment or MMP-9 deficiency did not change the action potential duration and conduction velocity (Supplementary Fig. S3).

### MMP-9 deficiency regulates RyR2 phosphorylation in mouse ventricular cardiomyocytes

**Figure 3:**
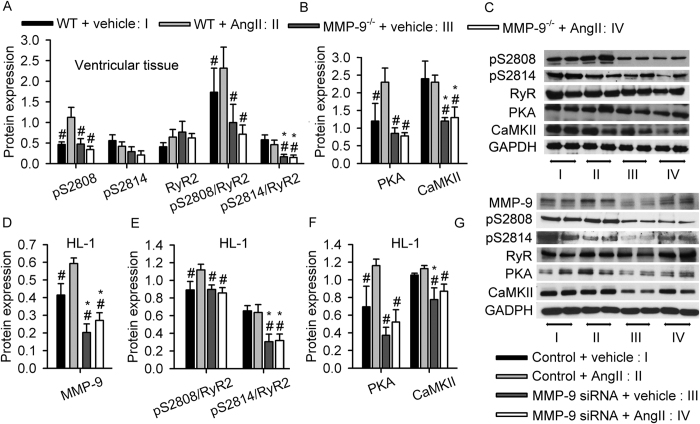
MMP-9 deficiency prevents RyR2 phosphorylation in cardiomyocytes. (**A**) Expression of RyR2 receptor phosphorylated at serine 2808 (pS2808) and 2814 (pS2814) in mouse ventricular tissue. The increased level of pS2808 in the WT mice after Ang II treatment that correlated with a ventricular arrhythmia attack was prevented in the MMP-9^−/−^ mice (n = 6, *P < 0.05 *vs.* WT + vehicle; ^#^*P* < 0.05 *vs.* WT + Ang II). (**B**) Corresponding changes in PKA and CaMKII in mouse ventricular tissue (n = 6, *P < 0.05 *vs.* WT + vehicle; ^#^*P* < 0.05 *vs.* WT + Ang II). (**C**) Representative Western blot showing phosphorylated RyR2 and PKA in mouse ventricular tissue. (**D** to **G**) MMP-9 expression was knocked down by siRNA in HL-1 cardiomyocytes (**D**), which prevented RyR2 hyperphosphorylation (**E**) and regulated PKA and CaMKII expression (**F**). (**G**) Representative Western blot in HL-1 cardiomyocytes. (**D**) to (**G**), (n = 4–6, *P < 0.05 *vs.* WT + vehicle, ^#^*P* < 0.05 *vs.* WT + Ang II). Data are presented as the mean ± SEM.

The expression levels of calcium-regulating genes including SERCA2a, RyR2, NCX1 and Cav1.2 in mouse ventricles did not differ between groups (Supplementary Fig. S4). We next hypothesized that calcium leakage via RyR2 might contribute to arrhythmogenesis^6^,^20^. Protein kinase A (PKA) and calcium/calmodulin-dependent protein kinase II (CaMKII) regulate RyR2 channel function via phosphorylation, which induces calcium leakage from the RyR2 channel on the SR and thus, ventricular arrhythmogenesis^21^. The RyR2 channel protein is phosphorylated by PKA at serine 2808 (pS2808) and by CaMKII at serine 2814 (pS2814)^21^. In both mouse ventricular tissue (Fig. 3A–C) and corresponding isolated ventricular cardiomyocytes (Supplementary Fig. S5), Ang II treatment increased PKA and pS2808 expression in WT mice, and this increase was correlated with inducible ventricular arrhythmia, although CaMKII and pS2814 levels were unaltered. MMP-9 deficiency consistently prevented PKA and pS2808 increases after Ang II treatment and reduced ventricular arrhythmia. These observations have been further replicated in HL-1 cells after treatment with MMP-9 siRNA (Fig. 3D–G).

### MMP-9 deficiency reduces Ca^2+^ leakage and irregular calcium transients in mouse isolated ventricular cardiomyocytes

**Figure 4:**
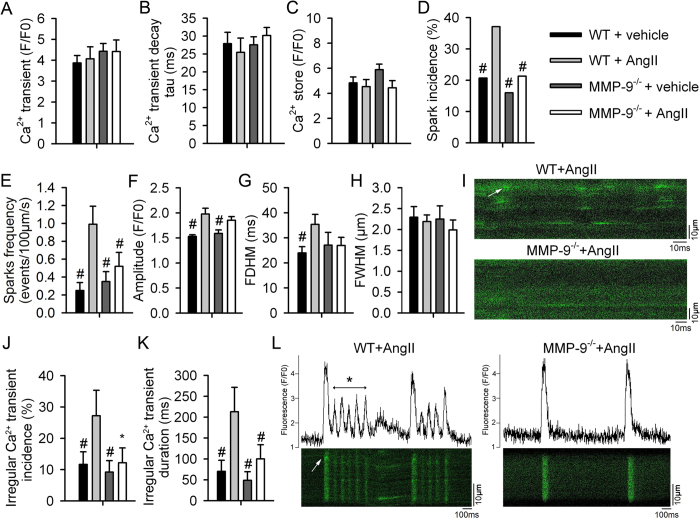
MMP-9 deficiency reduces Ca^2+^ leakage and irregular calcium transients in isolated ventricular cardiomyocytes. (**A** and **B**) Amplitude of Ca^2+^ transients and decay over time (n = 17–38, from 4–5 experiments, *P* = 0.73). (**C**) Amplitude of Ca^2+^ storage induced by caffeine (20 mM) (n = 11–17, from 4–5 experiments, *P* = 0.14). (**D** to **I**) A summary of Ca^2+^ sparks, including the incidence (**D**), frequency (**E**), amplitude (**F**), Ca^2+^ spark duration (FDHM, **G**), and width (FWHM, **H**), as well as a representative image (**I**). A calcium spark is denoted by a white arrow (n = 21–87, from 4–6 experiments, ^#^*P* < 0.05 *vs.* WT + Ang II). (**J** to **L**) A summary of irregular Ca^2+^ transients, including their incidence (**J**) and duration (**K**). Irregular, arrhythmia-like calcium transients (asterisk) early and delayed after depolarization were preceded by a calcium spark after ventricular pacing (arrow head), suggesting that calcium leakage might induce abnormal Ca^2+^ transients (**L**) in cardiomyocytes from WT mice after Ang II treatment. These irregular Ca^2+^ transients were absent in the MMP-9^−/−^ mice after Ang II treatment (n = 18–39, from 4–5 experiments, ^#^*P* < 0.05 *vs.* WT + Ang II, *P = 0.057 *vs.* WT + Ang II). Data are shown as the mean ± SEM.

Confocal microscopy revealed that MMP-9 deficiency did not affect cytosolic intracellular Ca^2+^ concentrations and decay times (Ca^2+^ transients, Fig. 4A and B) or SR Ca^2+^ stores (Fig. 4C) in mouse isolated ventricular cardiomyocytes. In contrast, Ang II treatment significantly increased the incidence and frequency of Ca^2+^ sparks, as well as spark amplitude and duration in WT mice, indicating increased Ca^2+^ leakage, although these changes did not have the same effect in the MMP-9^−/−^ mice (Fig. 4D–I). MMP-9 deficiency prevented calcium leakage via RyR2, thus corroborating the down-regulation of phosphorylated RyR2 and PKA and reduction of inducible ventricular arrhythmia^8^,^20^,^22^. In addition, the incidence and duration of spontaneous irregular calcium transients after Ang II treatment exhibited arrhythmic waveforms and resembled early or delayed afterdepolarizations, an effect that was abolished in the MMP-9^−/−^ cardiomyocytes (Fig. 4J–L). The observed Ca^2+^ sparks preceded irregular calcium transients, suggesting that a calcium leak triggered the arrhythmia and explaining why the ventricular arrhythmia vulnerability increased after Ang II treatment *in vivo* (Fig. 4L).

### MMP-9 inhibitors prevent abnormal calcium leakage in hiPSC-CMs

**Figure 5:**
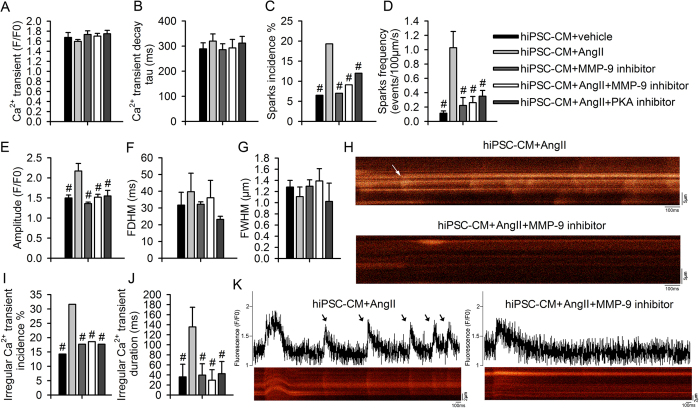
MMP-9 inhibition prevented Ca^2+^ leakage and irregular calcium transients in hiPSC-CMs. (**A** and **B**) Amplitude and decay of Ca^2+^ transients over time (n = 28–40, from 4 experiments, *P* = 0.48). (**C** to **H**) Summary of Ca^2+^ spark, including the incidence (**C**), frequency (**D**), amplitude (**E**), duration (FDHM, **F**), and width (FWHM, **G**), as well as a representative image (**H**). Ca^2+^ sparks (denoted by white arrows) after treatment with Ang II are prevented by an MMP-9 inhibitor (n = 50–145, from 6 to 8 experiments, ^#^*P* < 0.05 *vs.* WT + Ang II). (**I** to **K**) A summary of irregular Ca^2+^ transients, including their incidence (**I**) and duration (**J**), and a representative image (**K**). Irregular calcium transients were induced only in a high-Ca^2+^ (3.6 mM) Tyrode solution. Irregular, arrhythmia-like calcium transients, denoted by black arrows, were induced after treatment with Ang II and were prevented by MMP-9 or PKA inhibition (n = 33–73, from 3 to 5 experiments, ^#^*P* < 0.05 *vs.* WT + Ang II). Data are shown as the mean ± SEM.

HiPSC-CMs are considered “a human model in a dish” to overcome translational hurdles stemming from the paucity of human cardiac tissue and differences between species^23^,^24^,^25^. hiPSC-CMs displayed distinct myocardial fibrils (Supplementary Fig. S6). The calcium transients, decay times (Fig. 5A and B) and spontaneous beating rates (Supplementary Fig. S7) did not differ after treatment with Ang II, an MMP-9 inhibitor (ab142180), or a PKA-specific inhibitor (H-89). Ang II treatment substantially increased the incidence, frequency, and amplitude of calcium sparks in hiPSC-CMs, and these changes were prevented by treatment with an MMP-9 inhibitor or PKA inhibitor (Fig. 5C to E). Spark duration and spatial width did not differ (Fig. 5F–H). Upon subjecting the hiPSC-CMs to high Ca^2+^ (3.6 mM)^23^,^26^, irregular calcium transients that mimicked arrhythmia were induced after Ang II treatment, although these transients were almost absent in control hiPSC-CMs and in those treated with MMP-9 or PKA inhibitors (Fig. 5I–K).

**Figure 6:**
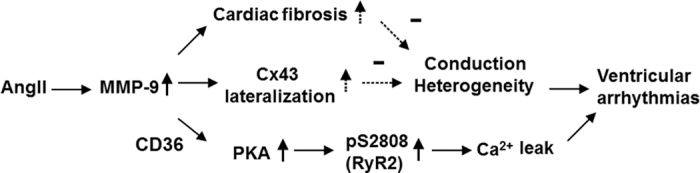
Mechanisms of MMP-9 inhibition to prevent ventricular arrhythmia. MMP-9 does not change collagen and Cx43 production but increases Cx43 lateralization. These minor changes might not significantly alter the conduction heterogeneity. In addition, MMP-9 can act as an intracellular signal regulator through the degradation of CD36 and increase calcium leakage, ventricular triggers, and arrhythmia through PKA and the hyperphosphorylation of RyR2.

We hypothesized that the activation of PKA and RyR2 phosphorylation was attributed to CD36-dependent inhibition of the cAMP/PKA signaling cascade. AngII treatment decreased the cardiac expression of CD36 in the WT mice. MMP-9 deficiency prevented the decrease of CD36 after Ang II treatment (Supplementary Fig. S8). The application of activated MMP-9 recombinant proteins to cardiomyocytes would increase MMP-9 cleaved 35-kDa fragments of CD36, confirming that CD36 is a substrate of MMP-9^27^. Further, the increased expression of CD36 by a PPARα-specific agonist (fenofibrate) would prevent the change of incidence, frequency, and amplitude of calcium sparks in hiPSC-CMs after AngII treatment (Supplementary Fig. S8). The mechanisms of MMP-9 inhibition in preventing ventricular arrhythmia are summarized in Fig. 6.

### Doxycycline reduced ventricular arrhythmia in a mouse model

Doxycycline was administered to our animal model at a clinical dosage using the same administrative route (nasogastric tube) applied in humans^28^. Feeding mice with doxycycline decreased MMP-9 activity and reduced ventricular arrhythmia after Ang II treatment (Supplementary Fig. S9). The incidence, duration and threshold of ventricular arrhythmia were all reduced in mice fed doxycycline compared with the vehicle control after Ang II treatment.

## Discussion

The present study extended our understanding of the role of MMP-9 in the pathogenesis of ventricular arrhythmia. High MMP-9 levels were causally linked to ventricular arrhythmia in mouse models, and mice genetically deficient in MMP-9 presented a profoundly decreased vulnerability to ventricular arrhythmia. The pleiotropic effects employed by MMP-9 inhibition to prevent ventricular arrhythmia extend beyond ECM regulation and gap junction remodeling. Furthermore, the distinct mechanism of MMP-9 deficiency prevents calcium leakage via PKA and its associated phosphorylated RyR2 receptors in both hiPSC-CMs and mouse cardiomyocytes. The oral administration of doxycycline successfully prevented ventricular arrhythmia in the mouse model, thus demonstrating the potential translational significance of MMP-9 inhibition.

MMP-9 activation in the ventricle increased the incidence of ventricular arrhythmia in our mouse model, whereas the down-regulation of MMP-9 by gene modification or pharmacological inhibition significantly reduced the incidence of ventricular arrhythmia. A dose-dependent reduction of ventricular arrhythmia was observed in comparisons of WT and MMP-9 heterozygous or homozygous knock-out mice. In addition, irregular arrhythmia-like calcium transients were inhibited by an MMP-9 inhibitor in a human cardiomyocyte model. Overall, these results suggest that MMP-9 is causally linked to ventricular arrhythmogenesis. Abnormal calcium leakage and transients in the hiPSC-CMs were correlated with a clinical arrhythmic phenotype^23^,^24^,^25^,^29^. We extended the benefits of hiPSC-CMs to study the arrhythmic mechanisms of MMP-9 *in vitro* following pathological stress. Increased MMP-9 expression has been observed in patients with heart failure^13^,^15^, although the functional impacts are difficult to study in humans because isolated human cardiomyocytes are unavailable. However, irregular calcium transients and sparks mimicking clinical arrhythmia were inhibited by a specific MMP-9 inhibitor in hiPSC-CMs, which satisfied our desire to validate the functional impact and pathogenesis of MMP-9 in human cardiomyocytes.

MMP-9 inhibition might exert pleiotropic effects to prevent ventricular arrhythmia, including reduced cardiac fibrosis, gap junction remodeling and calcium homeostasis. In hearts, MMP-9 is primarily secreted by leukocytes, fibroblasts and myofibroblasts, and it is functional in its secreted form in the ECM and in its anchored form on the cell membrane^9^,^10^,^11^. Our work suggested that MMP-9 deficiency did not prevent the increase of collagen production and only increased Cx43 lateralization. High levels of Cx43 reduction, lateralization, and fibrosis were necessary to create conduction heterogeneity for reentry^30^,^31^. A substantial reduction of fibrosis, Cx43 reduction, and lateralization might also be necessary to reverse the ventricular conduction. Conduction velocity of mice ventricles did not change after AngII treatment in the present study. Therefore, fibrosis and Cx43 lateralization was not linked to electrical conduction and ventricular arrhythmias in the present study (Fig. 6). Supported by functional and molecular studies of human and animal cardiomyocytes, we also found that myocardial MMP-9 can act as an intracellular signal regulator and increase calcium leakage, ventricular triggers and arrhythmia. This function is distinct from the well-known role of MMP-9 as a proteolytic enzyme in the ECM and has not been proposed for other MMPs^10^,^11^,^19^,^32^,^33^,^34^,^35^,^36^.

How MMP-9 activates PKA and RyR2 phosphorylation may be attributed to CD36-dependent inhibition of the cAMP/PKA signaling cascade. CD36, a fatty acid transport protein, inhibits myocardial PKA activity and cAMP levels through tyrosine kinase-dependent mechanisms such as JNK or p38^37^,^38^,^39^. CD36 is recently reported as an MMP-9 substrate^27^. As MMP-9 could proteolytically degrade CD36, increased MMP-9 levels would decrease CD36 and its inhibition of PKA^27^. Therefore, PKA activity and RyR2 phosphorylation is subsequently upregulated, leading to ventricular arrhythmias. Increased RyR2 phosphorylation can result in a higher probability of RyR pore opening^8^,^40^. An increase in calcium sparks activates an arrhythmogenic depolarizing inward Na^+^/Ca^2+^ exchange current, which causes delayed afterdepolarizations and triggers ventricular arrhythmias^8^,^40^. PKA increases the phosphorylation of RyR2, leading to a loss of calstabin2 from the macromolecular channel complex, and increases calcium leakage, ventricular arrhythmia, and sudden cardiac death^8^,^41^,^42^. In diseased human cardiomyocytes from an aortic stenosis-related hypertrophic heart, the activation of RyR2 by PKA also increased calcium sparks^43^. PKA might also change CREB-related transcriptional modification, one of the regulators of the pathogenesis of cardiac hypertrophy^44^. However, we did not observe any differences in CREB or phosphorylated CREB expressions (data not shown). Rather, our results showed that MMP-9 and PKA inhibitors prevent calcium sparks and irregular calcium transients, which also prevented ventricular arrhythmia in mice and our modeled human hiPSC-CMs.

We observed that both levels of pS2808 and pS2814 could be regulated by MMP-9 deficiency. However, the increased levels of pS2808 and PKA were associated with ventricular arrhythmia induction and calcium leakage in our animal and cardiomyocyte models. The levels of pS2814 and CaMKII did not increased in WT mice with ventricular arrhythmias after AngII treatment. Therefore, pS2814 and CaMKII are not likely the cause of ventricular arrhythmias in our experiment. In addition to the roles of MMP-9 in activating PKA, MMP-9 might also regulate the expression of CaMKII. The inhibition of insulin-like growth factor (IGF-II) would decrease the expression of CaMKII in cardiomyocytes^45^. MMP-9 deficiency prevents the proteolysis of protein complexes formed by IGF-II and its binding proteins, which consequently decreases the release of free IGF-II and the expression of CaMKII^45^,^46^. The physiological consequence of S2814 and CaMKII down-regulation in MMP-9^−/−^ mice remains unclear.

Hypertrophy does not occur in MMP-9^−/−^ mice after AngII treatment, which suggests that MMP-9 is an important factor in AngII-induced hypertrophy. It has been suggested that cardiac hypertrophy and calcium homeostasis in MMP-9 deficiency could be independently regulated^47^,^48^. The protein kinase A is modulated by a family of A-kinase anchoring proteins (AKAPs), which form multiprotein complexes and enable segregated cAMP signaling events to occur in defined cellular compartments. AKAP-Lbc mediated PKA-associated hypertrophic signal pathways through NFAT and MEF2^47^. However, AKAP-12 and AKAP-18 mediated PKA-associated calcium homeostasis^47^. The dysregulation of calcium homeostasis could be an independent pathway rather than secondary to cardiac hypertrophy.

Current medical treatments for ventricular arrhythmia remain limited or harmful^5^. Doxycycline has been used for MMP-9 inhibition in patients with an abdominal aneurysm in a clinical trial^28^. Doxycycline prevented ventricular arrhythmia, suggesting MMP-9 inhibition as a potential therapy. The potential benefits of using MMP-9 inhibitors to treat ventricular arrhythmia are manifold. Patients with ventricular arrhythmia commonly have cardiac fibrosis and impaired heart function^1^, and most antiarrhythmic drugs could not be used in patients with cardiac contractile dysfunction^5^. In the present study, MMP-9 inhibition reduced cardiac fibrosis and Cx43 lateralization and exerted a neutral effect on the calcium transients and ejection fraction, thus suggesting potential benefits for patients with cardiac dysfunction. Furthermore, doxycycline did not change the spontaneous heart rate, whereas anti-arrhythmic drugs suppressed sinus node function, leading to bradycardia and syncope. Nevertheless, the efficiency of MMP-9 inhibitors must be confirmed in future large-animal or clinical studies.

## Methods

### Mouse model of ventricular arrhythmia

We used a mouse model of ventricular arrhythmia induced by Ang II infusion^49^,^50^,^51^. Mice were anesthetized using Zoletil 50 (5 mg/kg) with Xylazine (Ropum). Through a mid-scapular incision, we spread the subcutaneous tissue to create a pocket and inserted a filled pump into the pocket. Only male mice aged 12–15 weeks were used. WT littermates, MMP-9 homozygous (MMP-9^−/−^, Cg-MMP9^tm1Tvu/J^; Jackson Laboratory, Bar Harbor, ME, USA) and heterozygous knock-out mice (MMP-9^+/−^) in an FVB background were treated with Ang II (2.0 mg/kg per day, Sigma-Aldrich, St. Louis, MO, USA) or saline (vehicle) administered by a subcutaneous ALZET micro-osmotic pump (model 1002) for 14 days (n = 10 for each group). The heart rate and blood pressure of the mice were measured using a noninvasive computerized tail-cuff system (BP98A, Softron, Tokyo, Japan) before and 2 weeks after pump implantation^52^. An EP study was performed 2 weeks after Ang II infusion. Echocardiography was performed before the EP study to determine the left ventricular ejection fraction. Multiple physiological variables were evaluated in an open-label design.

### Oral administration of doxycycline

The WT littermates treated with Ang II or vehicle delivered via a subcutaneous ALZET osmotic pump were randomized into two groups (i.e., oral feeding with doxycycline or not). Doxycycline was fed once daily (0.12 mg/g/day) using a nasogastric tube beginning on the day of the osmotic pump implantation and continuing for 14 days. An EP study was performed 2 weeks after Ang II infusion. In total, 4 groups were established to test the potency of doxycycline for preventing ventricular arrhythmia (Group I: WT with vehicle pump; Group II: WT with vehicle pump and doxycycline feeding; Group III: WT with Ang II pump; and Group IV: WT with Ang II pump and doxycycline feeding).

### Mouse EP study

After administration of an isoflurane anesthetic, the mice were mechanically ventilated at a controlled temperature (37 °C ± 0.5 °C). The surface ECG (channel I, II, and aVF) was recorded by needle electrodes inserted subcutaneously in the *limbs. A m*ini-thoracotomy was performed in the right parasternal area, and epicardial recording and stimulation electrodes were positioned on the right ventricle. An EP study was performed using an epicardial approach with mini-electrodes. The pacing thresholds (in milliamperes) were determined for the stimulation leads using 1.0-ms pulse widths at twice the diastolic capture threshold. The cardiac rhythm was continuously monitored and recorded, and all of the ECG frontal axes (P and QRS) and time intervals (PR, QRS, QT, and RR) were calculated for each animal. The effective refractory period of the right ventricle was determined using the ventricular extra-stimulation method (S1S2). Ventricular arrhythmia was induced by burst ventricular pacing (S1S1) down to a minimum coupling interval of 40 ms for ten cycles in each mouse. The duration of the induced ventricular arrhythmia episodes was calculated. After the EP study, the mouse hearts were removed for tissue, protein or RNA analysis. This study protocol was reviewed and approved by the Institutional Animal Care Committee of Taipei Veterans General Hospital and conformed to the NIH guidelines (Guide for the Care and Use of Laboratory Animals).

### Calcium imaging by confocal microscopy

Isolated ventricular cardiomyocytes or hiPSC-CMs in Tyrode’s solution were loaded with Ca^2+^ indicators (10 μM Fluo-3/AM for isolated ventricular cardiomyocytes; 2 μM Rhod-2 for hiPSC-CM; Calbiochem, San Diego, CA, USA) and incubated at room temperature for 30 min in the dark. The cells were repetitively scanned over 3-ms intervals for a total duration of 6s. Fluorescence imaging was performed with a laser scanning confocal microscope (Zeiss LSM 510, Carl Zeiss, Jena, Germany). The fluorescence density (F) was normalized to the baseline fluorescence (F_0_) to obtain reliable data and determine the transient [Ca^2+^]_i_ changes relative to the baseline values (F/F_0_) and to exclude variations in the fluorescence intensity because of different volumes of injected dye. The transient [Ca^2+^]_i_ and peak systolic and diastolic [Ca^2+^]_i_ were measured during a 1-Hz field stimulation for 10 ms at twice the threshold strength in square wave pulses^53^. Ca^2+^ sparks were detected using the line-scan mode along a line parallel to the longitudinal axis of a single ventricular cardiomyocyte, thus avoiding the nucleus. Each line was composed of 512 pixels. Ca^2+^ sparks were defined as an increase in the signal mass of <6 μm. The Ca^2+^ sparks were analyzed using SparkMaster and validated by the authors using the signal mass criteria. The spark amplitude (ΔF/F0), full duration at half maximal amplitude (FDHM, ms), full width at half maximal amplitude (FWHM, μm), and Ca^2+^ spark incidence during the diastolic phase of the ventricular cardiomyocytes were analyzed.

Others detailed methods and statistical analysis are described in the online data supplement.

### Statistical analysis

Data are presented as the mean ± SEM. Student’s *t*-test was used to identify differences between two groups when appropriate. Comparisons between multiple groups were performed with a two-way ANOVA (Holm-Sidak post hoc analysis) or a one-way ANOVA (LSD post hoc test) when appropriate. Comparisons of categorical data were performed using a Chi-squared test. A *P*-value < 0.05 was considered statistically significant. PSAW SPSS 18.0 was used for the statistical analysis.

## Additional Information

**How to cite this article**: Weng, C.-H. *et al*. Pleiotropic Effects of Myocardial MMP-9 Inhibition to Prevent Ventricular Arrhythmia. *Sci. Rep.* **6**, 38894; doi: 10.1038/srep38894 (2016).

**Publisher's note:** Springer Nature remains neutral with regard to jurisdictional claims in published maps and institutional affiliations.

## Supplementary Material

Supplementary Information
