# Supplementary material for: Pleiotropic Effects of Myocardial MMP-9 Inhibition to Prevent Ventricular Arrhythmia
Source: Sci Rep. 2016 Dec 14;6:38894. doi: 10.1038/srep38894 (PMC5155273; doi:10.1038/srep38894)
Supplement: Supplementary Information [file srep38894-s1.doc]

**Pleiotropic Effects of Myocardial MMP-9 Inhibition to Prevent Ventricular Arrhythmia**

Ching-Hui Weng, Fa-Po Chung, Yao-Chang Chen, Shien-Fong Lin, Po-Hsun Huang, Terry B.J. Kuo, Wei-Hsuan Hsu, Wen-Cheng Su, Yen-Ling Sung, Yenn-Jiang Lin, Shih-Lin Chang, Li-Wei Lo, Hung-I Yeh, Yi-Jen Chen, Yi-Ren Hong, Shih-Ann Chen, Yu-Feng Hu

**Supplementary Material**

**Materials and Methods**

**Gelatin zymography**

We performed zymography using mouse peripheral blood and homogenized protein from mouse hearts. Blood and homogenized protein were collected from mouse ventricles as follows: mouse blood was collected using the facial vein technique and centrifuged at 3,000 rpm for 10 min at 4°C to obtain serum. Whole hearts were homogenized in lysis buffer containing 50 mM Tris, pH 7.6, 150 mM NaCl, 0.1% SDS, 0.5% sodium deoxycholate, and 1% Triton X-100 without protease or phosphatase inhibitors. The homogenates were centrifuged at 13,000 rpm for 20 min at 4°C, and the supernatants were collected. Protein concentrations were determined using a Pierce BCA protein assay kit (Thermo Fisher Scientific, Waltham, MA, USA).

A total of 50 µg of non-denatured protein was mixed with 2X Tris-Glycine SDS sample buffer [0.5 M Tris-HCl (pH 6.8), 20% glycerol, 10% (w/v) SDS, and 0.1% bromophenol blue], incubated at room temperature for 10 min, and run on a 7.5% polyacrylamide gel containing 0.1% gelatin (BD Difco™) in an electrophoresis unit. The gel was washed twice for 30 min with zymogram renaturing buffer (2.5% Triton X-100) to remove the SDS and then placed in zymogram developing buffer (50 mM Tris-HCl, 200 mM NaCl, 5 mM CaCl_2_, and 0.02% Brij 35) for 16 h at 37°C. After incubation, the gel was stained with 0.5% Coomassie blue R-250 (in 45% methanol, 10% acetic acid and 45% ddH_2_O) for 30 min and then destained with Coomassie blue R-250 destaining solution (50% methanol, 10% acetic acid and 40% ddH_2_O). MMP-9 and MMP-2 protease activity were shown as clear bands against a blue background. The bands were quantitatively analyzed using AlphaEaseFC 4.0 software.

**Histological analysis and immunofluorescence staining of mouse hearts**

Whole hearts were fixed in 10% neutral buffered formalin, embedded in paraffin wax, and cut into 4-μm sections. Sections were stained with hematoxylin and eosin (H&E) to examine any inflammation in and the histopathology of the cardiac tissue and were then stained by Masson’s trichrome stain to identify cardiac fibrosis ^1^. Fibrotic areas and the interventricular septal wall thickness at the mid-ventricular level of the heart below the papillary muscle were measured using Image-Pro Plus 6.0 software. Cardiac inflammation was graded by two independent researchers using a previously established method (0: no inflammatory infiltrates, 1: <5% of the cross section involved [mild], 2: 5% to 10% of the cross section involved [moderate], 3: 10% to 25% of the cross section involved [moderate], and 4: >25% of the cross section involved [severe]) ^2^. For immunofluorescence staining, 4-μm paraffin sections were deparaffinized, rehydrated, antigen unmasked, and blocked in 3% bovine serum albumin solution. Antigen retrieval was performed using 0.05% trypsin solution with 0.1% CaCl_2_ at 37°C for 30 min. The sections were then incubated with the appropriate primary antibody, PE-conjugated troponin T (1:100, BD), Cx43 polyclonal antibody (1:400, Merck), or MMP-9 polyclonal antibody (1:100, Abcam), and Alexa Fluor-conjugated secondary antibodies (1:400, Thermo Fisher). Nuclei were counterstained with antifade reagent containing 4,6-diamidino-2-phenylindole (DAPI, Molecular Probes). Sections were imaged using a confocal laser scanning microscope (FV10i, Olympus).

**Real-time polymerase chain reaction (Real-time PCR)**

Total RNA was extracted from mouse ventricular tissue using an RNeasy® Mini Kit (QIAGEN, Venlo, Netherlands). cDNA was synthesized using a SuperScript® III First-Strand Synthesis System (Invitrogen, Carlsbad, CA, USA). Real-time quantitative PCR was performed using a Roche LightCycler® 480 Real Time PCR system with SYBR Green Master Mix. The primers for the genes of interest [collagen I, collagen III, L-type calcium channel (Cav1.2), sarcoplasmic reticulum Ca^2+^ ATPase (SERCA2a), sodium calcium exchanger (NCX1), and ryanodine receptor2 (RyR2)] are listed in Supplementary Table S4. GAPDH was used as an internal control.

**Western blot analysis**

Mouse ventricular tissue, HL-1 cells or isolated mouse ventricular myocytes were homogenized in lysis buffer containing 50 mM Tris (pH 7.6), 150 mM NaCl, 0.1% SDS, 0.5% sodium deoxycholate, 1% Triton X-100, and protease and phosphatase inhibitors to retrieve the protein homogenates. The supernatants were collected for Western blot analysis after the protein homogenates were centrifuged at 13,000 rpm for 20 min at 4°C. The protein concentrations were determined as described above. Equal amounts of denatured protein (30 µg/lane) were separated on 10% polyacrylamide gels by SDS-PAGE or NuPAGE 3-8% Tris-Acetate gradient gels (for RyR2, pS2808, pS2814) (Invitrogen, Carlsbad, USA). The proteins were transferred to polyvinylidene difluoride (PVDF) membranes (Millipore, Billerica, MA, USA) and blocked with 5% non-fat dry milk. The membranes were incubated overnight with primary antibodies followed by HRP-conjugated secondary anti-mouse and anti-rabbit antibodies for 1 h at room temperature. Protein expression was examined using ECL Western blotting detection reagents (Millipore, Billerica, MA, USA) and analyzed using AlphaEaseFC 4.0. The primary antibodies included rabbit anti-MMP-9 (1:1000, Abcam), rabbit anti-connexin43 (1:1000, Sigma), rabbit anti-NOX2/gp91phox (NOX2, 1:1000, Abcam), rabbit anti-NADPH oxidase 4 (NOX4, 1:1000, Abcam), rabbit anti-SERCA2 (1:1000, Abcam), mouse anti-protein kinase A (PKA-RIIβ, 1:1000,BD Biosciences), mouse anti-Ca^2+^/calmodulin protein kinase II total (CaMKII, 1:1000, BD Biosciences), rabbit anti-ryanodine receptor2 (RyR2, 1:1000, Alomone), rabbit anti-pRyR2808 (1:1000, Abcam), rabbit anti-pRyR2814 (1:500, Badrilla), rabbit anti-CREB (1:1000, Cell Signaling), rabbit anti-phospho-CREB (1:1000, Cell Signaling), rabbit anti-CD36 (1:1000, Abcam), mouse anti-actin (1:1000, Abcam), and mouse anti-GAPDH (1:1000, Thermo Fisher Scientific).

**Isolation of ventricular cardiomyocytes**

Ventricular cardiomyocytes were isolated according to published protocols, with modifications ^3^. Briefly, mice were anesthetized using Zoletil 50 (5 mg/kg) with Xylazine (Ropum) and heparin (5000 units). The hearts were removed by midline thoracotomy and cannulated through the aorta on a Langendorff Apparatus with pressure perfusion at 37°C. The heart was first perfused with normal Tyrode’s solution (NaCl 137 mM, MgCl_2_ 0.5 mM, CaCl_2_ 1.8 mM, KCl 5.4 mM, HEPES 10 mM and glucose 10 mM, pH 7.4) for 10 min and then digested with Ca^2+^-free solution containing 1 mg/mL collagenase (Type I, Sigma) and 0.06 mg/mL proteinase (type XIV, Sigma). The Ca^2+^-free solution contained NaCl 120 mM, KCl 5.4 mM, KH_2_PO_4_ 1.2 mM, MgSO_4_ 1.2 mM, HEPES 6 mM, taurine 10 mM, and glucose 10 mM (pH 7.4). After perfusion, the heart was removed from the cannula, cut into small pieces, gently triturated with a plastic transfer pipette, and filtered through a mesh cell collector. Rod-shaped cells with clear striations and without granulation were used for all experiments. The dissociated cells could be stored for up to 6 h at room temperature in Tyrode’s solution.

**MMP-9 siRNA transfection**

HL-1 cardiomyocytes were cultured and maintained in Claycomb medium. At 80% confluency, 50 nM MMP-9 siRNA (Ambion®, Invitrogen) or 50 nM negative control siRNA (Ambion®, Invitrogen) was transfected into the HL-1 cardiomyocytes for 6 h using Lipofectamine 2000 transfection reagent (Invitrogen) in Opti-MEM® I medium. After incubation at 37°C for 48 h, we further treated the cells with vehicle (water) and Ang II (1 μM) for 24 h, and then the proteins were extracted for Western blot analysis. The experiment included 4 groups: Group I: control siRNA with vehicle; Group II: control siRNA with Ang II; Group III: MMP-9 siRNA with vehicle; and Group IV: MMP-9 siRNA with Ang II.

**Cardiomyogenic differentiation of hiPSCs**

HiPSC-differentiated cardiomyocytes (reproCELL, Kanagawa, Japan) were purchased and prepared according to the company’s instructions. Ethical approval was granted by the Institutional Review Board of the Veterans General Hospital, Taipei, Taiwan and conformed to the declaration of Helsinki. Briefly, the cells (3.4×10^5^ cells/tube) were thawed and prepared to a final concentration of 0.5×10^5^ cells/mL by adding medium (day 0). A cell suspension of 1.0×10^4^ cells/well was added to each 96-well plate, where it was expected to form a loose cell clump within 3 days. At day 3, the cell clumps were mechanically dissociated into 5-10 small microclusters and transferred into a 35-mm glass-bottom dish after coating. The attached microclusters formed a thin layer of cells that began to beat 2 to 3 days after transfer ^4^. The culture medium was changed every other day. Six days after the cells were transferred to the glass-bottom dish (day 9), they were pretreated with the MMP-9 inhibitor (10 nM, ab142180, Abcam), the PKA inhibitor (2 μM, H-89) or vehicle. Ang II (1 μM) was added on day 10, one day after the MMP-9 or PKA inhibitor, and a calcium assay was performed 24 h after Ang II treatment (day 11) ^4^.

**Calculation of Cx43 lateralization**

Cx43 lateralization was analyzed using a method based on quantification of the angle formed between the local longitudinal cell axis and the main axis of individual Cx43 clusters. The normal Cx43 gap-junctional distribution consists of a transversely oriented pattern consistent with the positions characteristic of the intercalated disks of the normal ventricular myocardium. The lateralization of Cx43 gap junctions was distributed mainly along the lateral interfaces between myocytes, appearing as longitudinally oriented arrays. The ratio of the fluorescence intensity of the lateral localization and total fluorescence intensity (transverse plus cell end localization in longitudinally oriented cardiomyocytes) of the Cx43 was used to indicate the lateralization. The tissue section (2 slides) from the mid-anterior and lateral wall of each mice ventricle (n=8-11 animals) was analyzed.

**Optical mapping of mice ventricles**

Langendorff-perfused mouse hearts were prepared and optical mapping techniques^5^ were used to study the left ventricle. The hearts were stained with 20 μL of the voltage-sensitive dye (Di-4-ANEPPS, 2mmol/L) for imaging membrane potential. Optical signals were processed with spatial (3 × 3 pixels Gaussian filter) and temporal (3 frames moving average) filtering. Optical recording was performed after 100 beats of stable pacing at pacing cycle length of 120ms. To study the epicardial ventricular conduction velocities, a pacing electrode was placed on the apical wall of the left ventricle. Action potential duration (APD) was measured at 70% of repolarization (APD70) of 4 consecutive beats of ≥4 ms.

**Supplementary Table S1. Electrocardiographic and electrophysiological parameters of the WT and MMP-deficient mice 2 weeks after treatment with Ang II or vehicle.**

|  | WT + vehicle | WT + Ang II | MMP-9^+/-^ + Ang II | MMP-9^-/-^ + vehicle | MMP-9^-/-^ + Ang II | *P-*value |
| --- | --- | --- | --- | --- | --- | --- |
| P wave duration (ms) | 14.6±0.8 | 17.6±1.1 | 16.8±0.6 | 15.6±1.0 | 18.0±0.8 | 0.61 |
| PR interval (ms) | 29.3±1.0 | 30.7±2.0 | 30.5±0.8 | 31.0±1.0 | 30.7±1.7 | 0.91 |
| QRS duration (ms) | 18.0±0.5 | 20.0±1.7 | 18.3±0.4 | 18.3±0.7 | 21.0±1.1 | 0.15 |
| QT interval (ms) | 42.7±3.4 | 40.9±2.4 | 45.2±1.5 | 40.0±2.2 | 44.1±2.4 | 0.54 |
| VERP (ms) | 24.0±3.4 | 32.0±2.9 | 27.0±3.0 | 23.0±3.0 | 21.5±3.2 | 0.14 |

WT: wild type, MMP-9^+/-^: heterozygous knock-out mice, MMP-9^-/-^: homozygous knock-out mice; n=10 for each group; VERP: Ventricular effective refractory period.

**Supplementary Table S**2. Comparisons of the characteristics of the WT and MMP-deficient mice 2 weeks after treatment with Ang II or vehicle.

|  | WT + vehicle | WT + Ang II | MMP-9^+/-^ + Ang II | MMP-9^-/-^ + vehicle | MMP-9^-/-^ + Ang II |
| --- | --- | --- | --- | --- | --- |
| Body weight (g) | 31.9±0.4 #§≠* | 27.3±0.3 §≠* | 25.3±0.4 | 29.9±0.6 ≠* | 24.4±0.4 |
| Heart rate (bpm) | 644.9±7.3 #≠* | 683.5±9.4 § | 707.9±15.8 | 628.8±13.2 ≠* | 689.3±9.2 |
| Systolic blood pressure (mmHg) | 108.1±1.3 #≠* | 153.6±2.1 § | 156.3±5.6 | 101.5±1.5 ≠* | 153.8±3.7 |
| Diastolic blood pressure (mmHg) | 73.5±1.4 #§≠* | 105.8±2.4 § | 112.0±5.9 | 64.5±1.5 ≠* | 106.2±2.5 |

WT: wild type, MMP-9^+/-^: heterozygous knock-out mice, MMP-9^-/-^: homozygous knock-out mice; n=10-25 for each group.

(#*P*<0.05 vs. WT + Ang II; §*P*<0.05 vs. MMP-9^-/-^+vehicle; ≠*P*<0.05 vs. MMP-9^-/-^ + Ang II; * *P*<0.05 vs. MMP-9^+/-^ + Ang II)

**Supplementary Table S**3. Mouse cardiac function by echocardiography.

|  | WT + vehicle | WT + Ang II | MMP-9^-/-^ + vehicle | MMP-9^-/-^ + Ang II | *P*-value |
| --- | --- | --- | --- | --- | --- |
| End systolic diameter (mm) | 20.00±2.64 | 16.60±1.16 | 20.66±2.13 | 19.57±2.55 | 0.63 |
| End diastolic diameter (mm) | 38.66±3.75 | 37.40±1.16 | 44.16±2.07 | 36.71±3.57 | 0.24 |
| Left ventricular ejection fraction (LVEF, %) | 0.71±0.08 | 0.79±0.02 | 0.77±0.03 | 0.71±0.04 | 0.41 |

WT: wild type, MMP-9^-/-^: homozygous knock-out mice; n=3-7 for each group

**Supplementary Table S4. Primers used for real-time polymerase chain reaction (Real-time** PCR).

| Transcript | Forward | Reverse |
| --- | --- | --- |
| Collagen I | CATGTTCAGCTTTGTGGACCT | GCAGCTGACTTCAGGGATGT |
| Collagen III | TCCCCTGGAATCTGTGAATC | TGAGTCGAATTGGGGAGAAT |
| L-type calcium channel (Cav1.2) | GCCAGCCCAGAAAAGAAACA | TTCATCCTCTTCCCCTGCAG |
| SERCA2a | TGAGACGCTCAAGTTTGTGG | CTTTTCCCCAACCTCAGTCA |
| Sodium calcium exchanger (NCX1) | CGAGACTGTGTCGAACCTGA | TCAGGGACCACGTAAACACA |
| Ryanodine receptor2 (RyR2) | AAGCCCTCACGACTAAAGCA | CCACCCAGACATTAGCTGGT |
| GAPDH | ACCCAGAAGACTGTGGATGG | CACATTGGGGGTAGGAACAC |

**Supplementary Figure S1. MMP-2 expression in mice after treatment with Ang II or vehicle.**

**
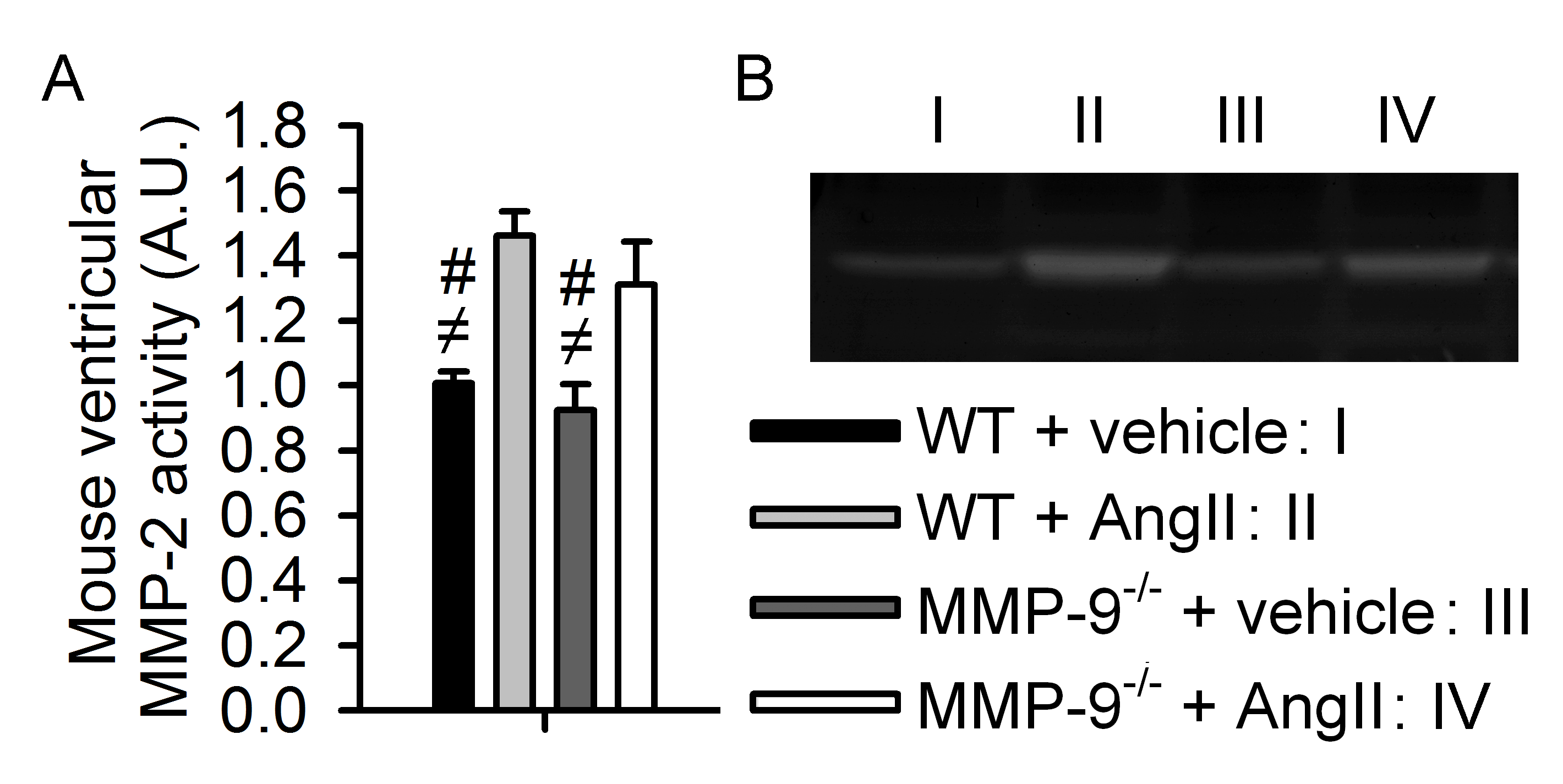
**

(A) MMP-2 enzymatic activity in ventricular tissue (n=4-6, #*P*<0.05 vs. WT + Ang II, ≠*P*<0.05 vs. MMP-9^-/-^ + Ang II). (B) Representative image of MMP-2 ventricular activity by zymography. Data are shown as the mean±SEM.

**Supplementary Figure S2. Inflammation and oxidative stress in MMP-9-deficient mice.**

**
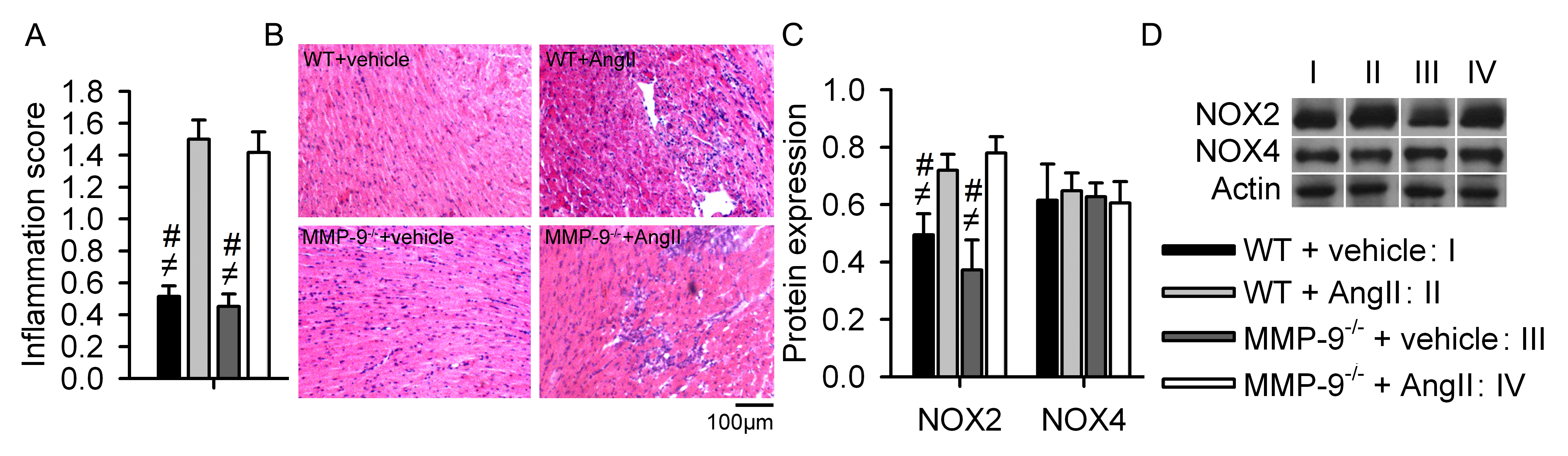
**

(A) Cardiac inflammation was determined by the severity of immune cell infiltration, and it increased after Ang II treatment in both the WT and MMP-9^-/-^ mice, whereas MMP-9 knock-out did not decrease the severity of Ang II-induced immune cell infiltration (n=4-6, #*P*<0.05 vs. WT + Ang II, ≠*P*<0.05 vs. MMP-9^-/-^ + Ang II). (B) Representative H&E staining in four experimental groups. (C) Expression of NADPH oxidases (NOX2 and NOX4, the main isoforms in myocytes) contributed to the production of reactive oxygen species (ROS) and oxidative stress. NOX2 expression increased in both WT and MMP9^-/-^ mice after Ang II treatment, and MMP-9 deficiency did not block Ang II-induced NOX2 increase (n=4-5, #*P*<0.05 vs. WT + Ang II, ≠*P*<0.05 vs. MMP-9^-/-^ + Ang II). NOX4 levels did not differ between the vehicle- and Ang II-treated animals for either the WT or MMP-9^-/-^ mice. (D) Representative Western blot showing NOX2 and NOX4 in the mouse ventricular tissue. Data are shown as the mean±SEM.

**Supplementary Figure S3. Action potential duration and conduction velocity in mice ventricles.**

**
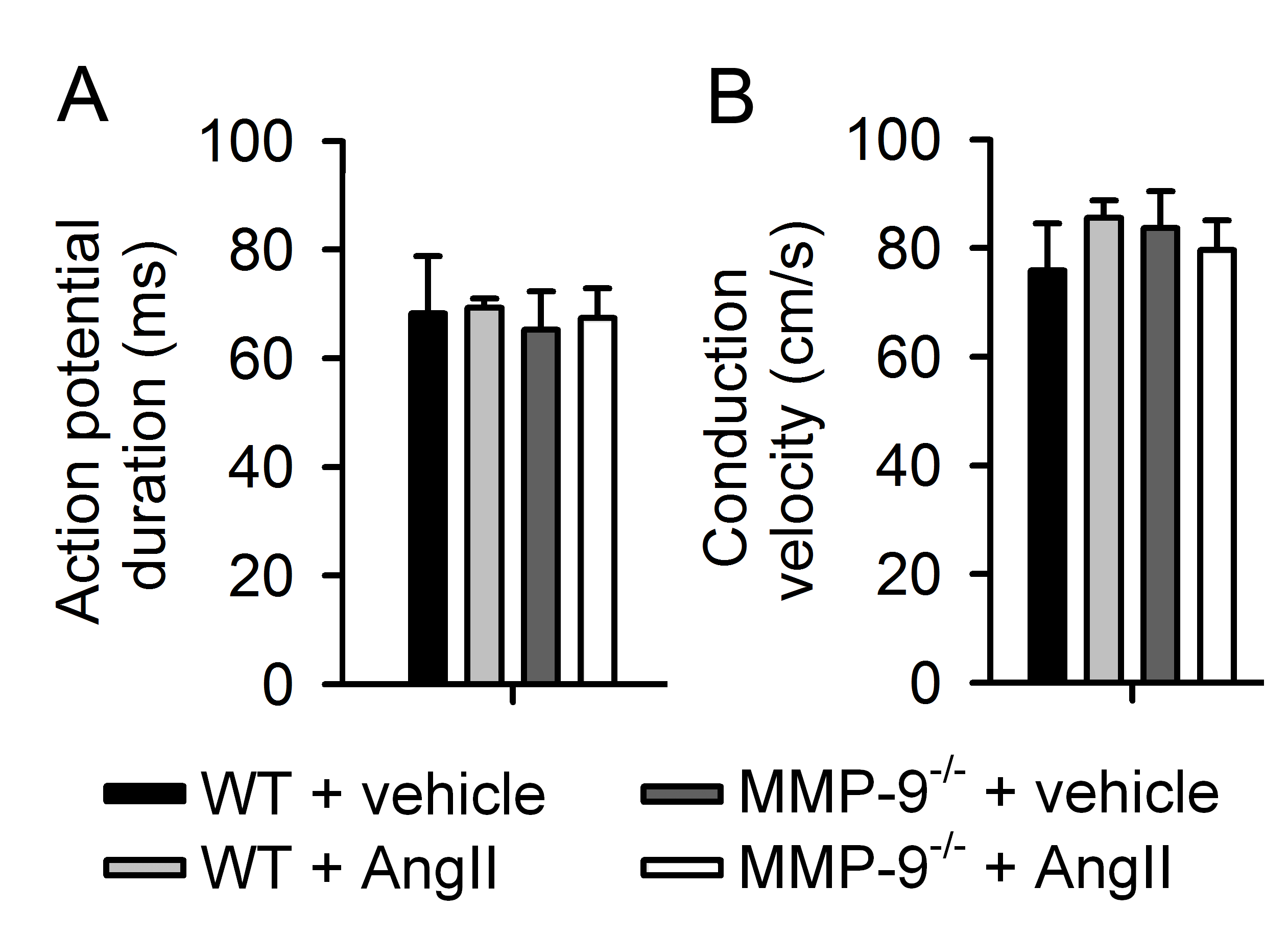
**

(A) Action potential duration (APD) was measured at 70% of repolarization (APD70) (n=3-5). (B) Conduction velocity of left ventricles (n=3-5). There was no difference of APD70 and conduction velocity between 4 groups. Data are shown as the mean±SEM.

**Supplementary Figure S4. Expression of calcium-regulating genes in mouse ventricular tissue.**

**
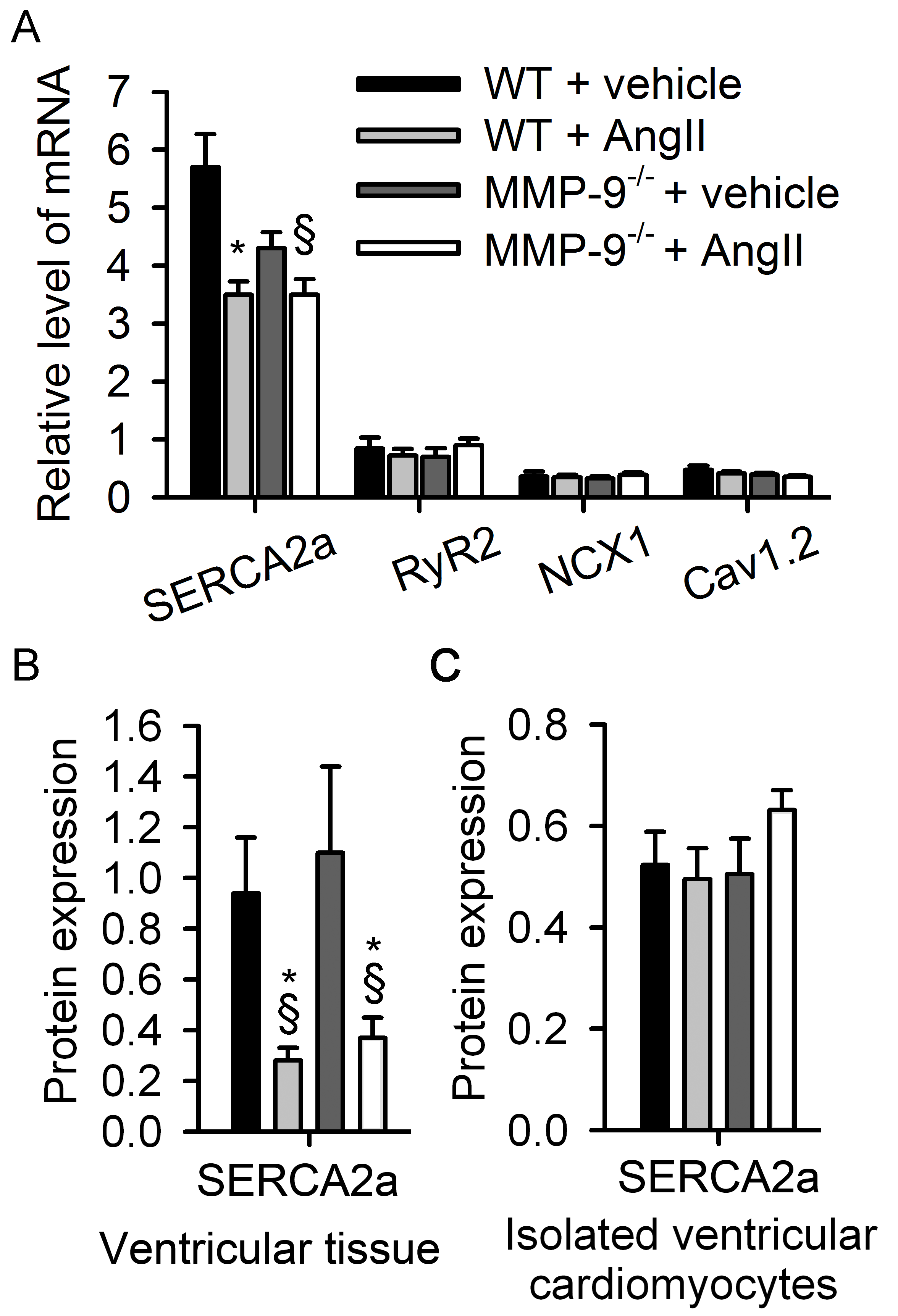
**

(A) SERCA2a mRNA was decreased in both WT and MMP-9^-/-^ mice after Ang II treatment (n=7-10, **P*<0.05 *vs.* WT + vehicle; §*P*<0.05 *vs.* MMP-9^-/-^ + vehicle), whereas RYR2, NCX1 and Cav1.2 mRNA expression levels did not differ between the groups (n=7-10). (B) SERCA2a protein expression in mouse ventricular tissue (n=8-10, **P*<0.05 *vs.* WT + vehicle; §*P*<0.05 *vs.* MMP-9^-/-^ + vehicle). (C) In isolated ventricular cardiomyocytes, the protein expression of SERCA2 by Western blot revealed that Ang II treatment did not result in a change in SERCA2a expression (n=4-5). Data are shown as the mean±SEM. In the ventricular tissue, Serca2A is expressed only in vascular smooth muscle and cardiomyocytes, but not in other cells such as endothelial cells or fibroblasts^6, 7^. Although AngII did not decrease Serca2A expression in isolated ventricular cardiomyocytes, AngII could suppress Serca2A expression through STAT3 in vascular smooth muscle cells. AngII treatment inhibits STAT3 in vascular smooth muscle cells^8^. The STAT3 binding site could be identified in the Serca2A promoter, which suggested the regulation of Serca2A expression by STAT3. Therefore, we attributed the decreased SERCA2a expression in mouse ventricles to vascular smooth muscle cells rather than to cardiomyocytes.

**Supplementary Figure S5. MMP-9 deficiency regulates RyR2 phosphorylation in mouse isolated ventricular cardiomyocytes.**


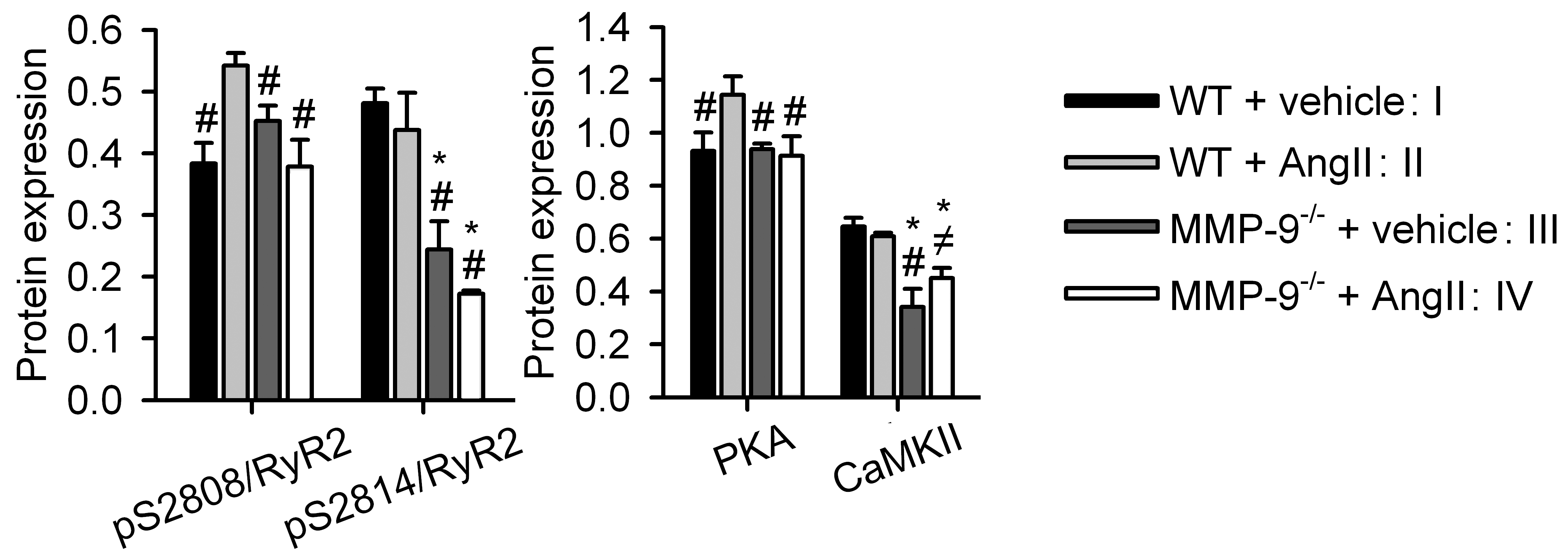


The ventricular cardiomyocytes from MMP-9^-/-^ and wild type littermate were isolated for the protein analysis. Summary of RyR2 phosphorylation in isolated ventricular cardiomyocytes, including phosphorylated RyR2, PKA and CaMKII expression, showed similar findings to those in the ventricular tissue (n=4-6, **P*<0.05 vs. WT + vehicle; #*P*<0.05 vs. WT + Ang II; ≠*P*=0.057 vs. WT + Ang II). Data are shown as the mean±SEM.

**Supplementary Figure S6. Myofibril structure in hiPSC-CMs.**


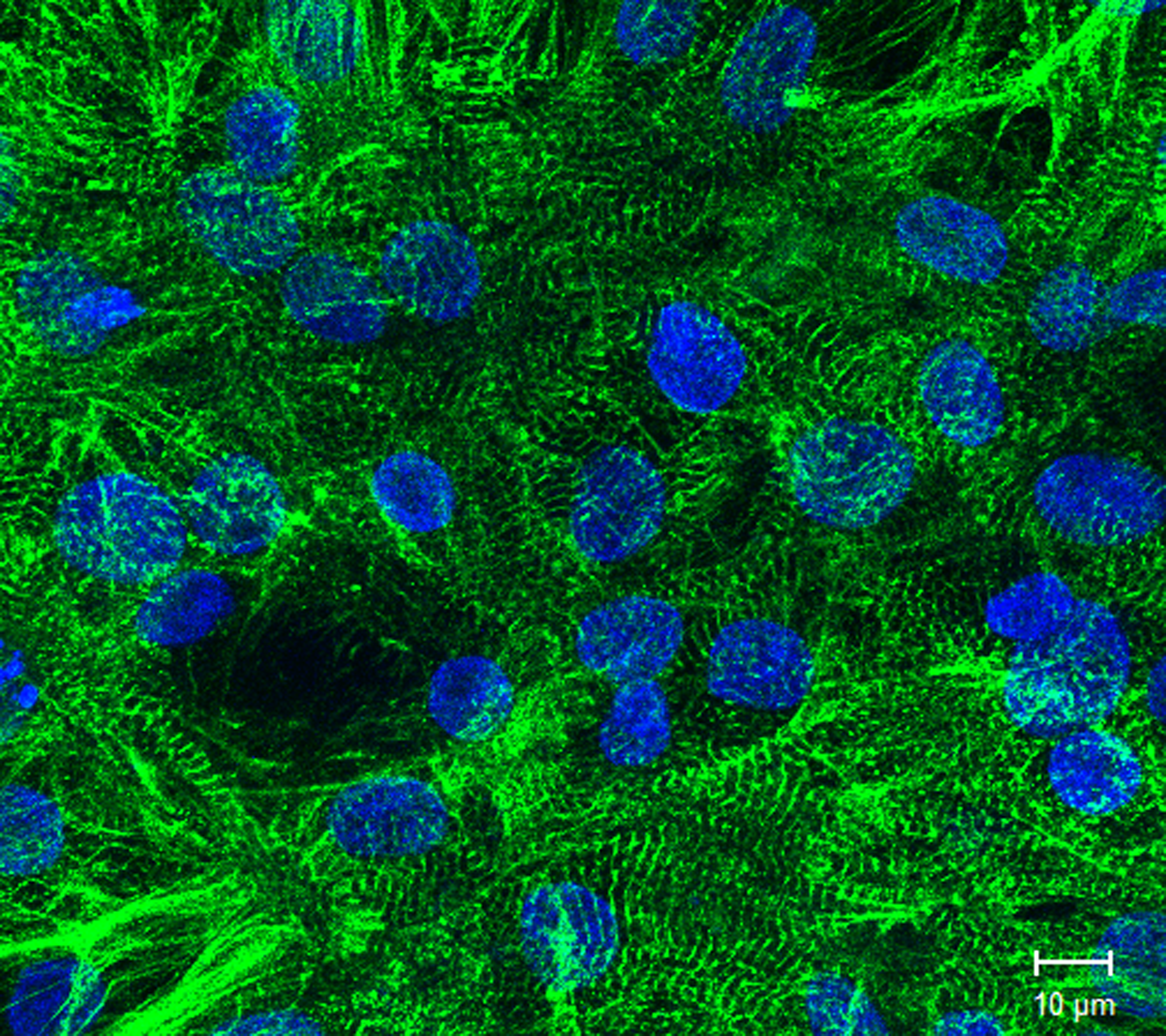


The myofibril structure in hiPSC-CMs (as revealed by α-sarcomeric actin [green]) was observed in 64.8±6.7% of the differentiated cells, which were observed to form monolayers and beat spontaneously. Nuclei are labeled in blue with DAPI.

**Supplementary Figure S7. Spontaneous beating rate in hiPSC-CMs.**

**
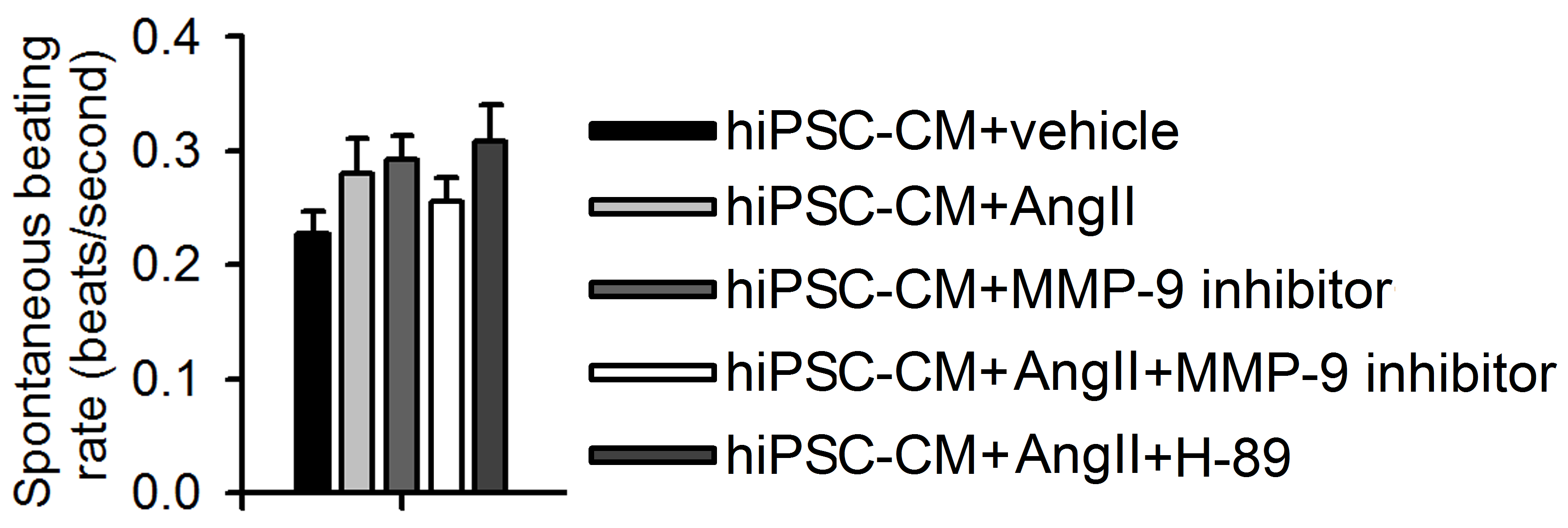
**

The spontaneous beating rate did not differ between groups (n=11-20, *P*=0.23). Data are shown as the mean±SEM. *P*-values were determined by a one-way ANOVA and LSD post-hoc test.

**Supplementary Figure S8. Role of CD36 in MMP-9 mediated calcium homeostasis**

**
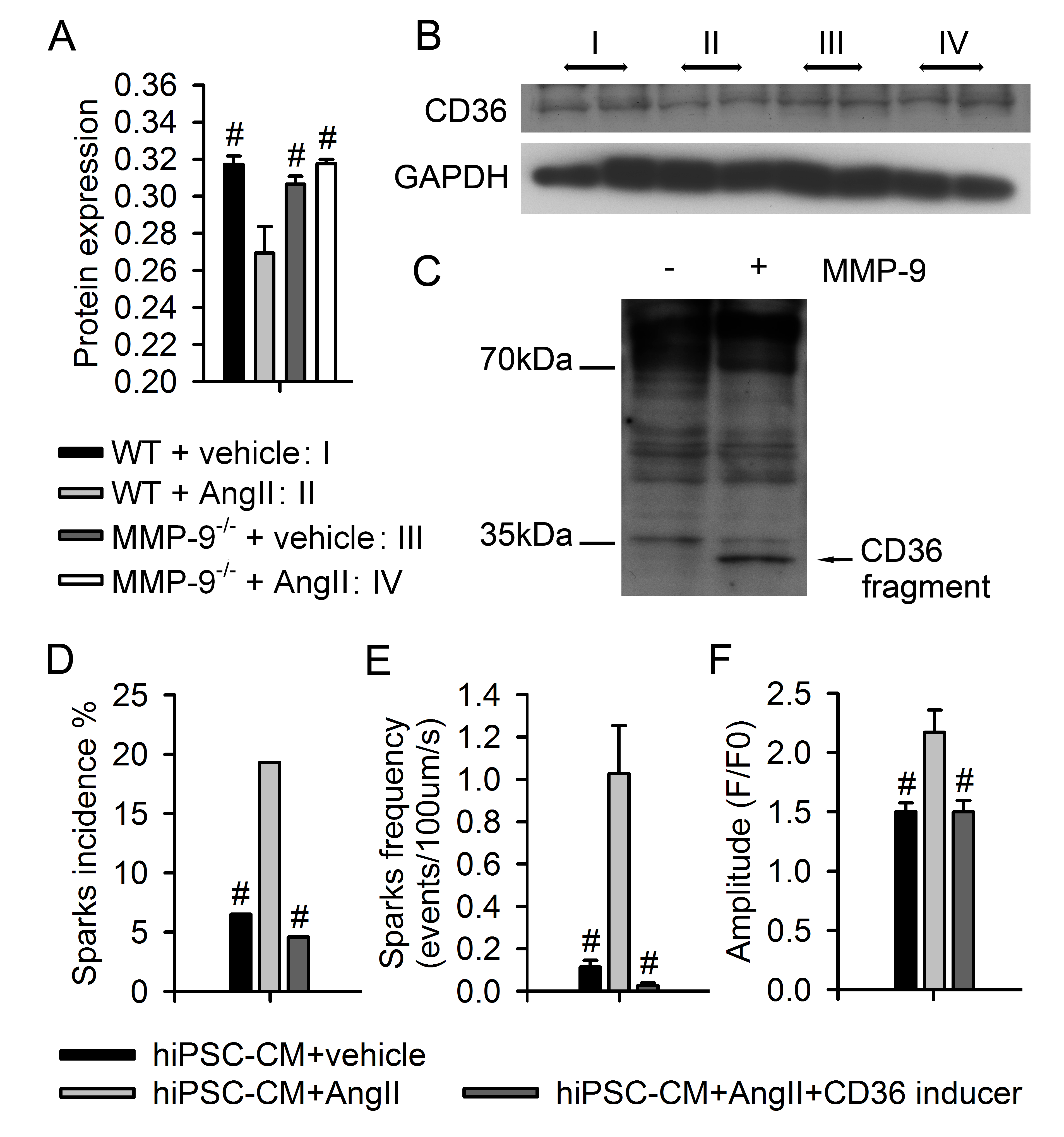
**

(A) Expression of CD36 in mouse ventricular tissue. The decreased levels of CD36 in the WT mice after Ang II treatment supported the degradation of CD36 by MMP-9, which was prevented by MMP-9 deficiency (n=4, *#P*<0.05 *vs.* WT + Ang II). (B) Representative Western blot showing CD36 in mouse ventricular tissue. (C) Immunoblotting of extracted protein from cardiomyocytes after the treatment of activated MMP-9 recombinant protein. A 35-kDa CD36 fragment (revealed using a CD36 antibody) is denoted by the arrow, indicating MMP-9 cleavage. (D to F) Summary of the Ca^2+^ spark, including the incidence (D), frequency (E), and amplitude (F) after the treatment of CD36 inducer (n=87-145, from 4 to 8 experiments, *#P*<0.05 *vs.* WT + Ang II). Data are shown as the mean±SEM.

**Supplementary Figure S9. Oral administration of doxycycline decreased MMP-9 activity and reduced ventricular arrhythmia.**

**
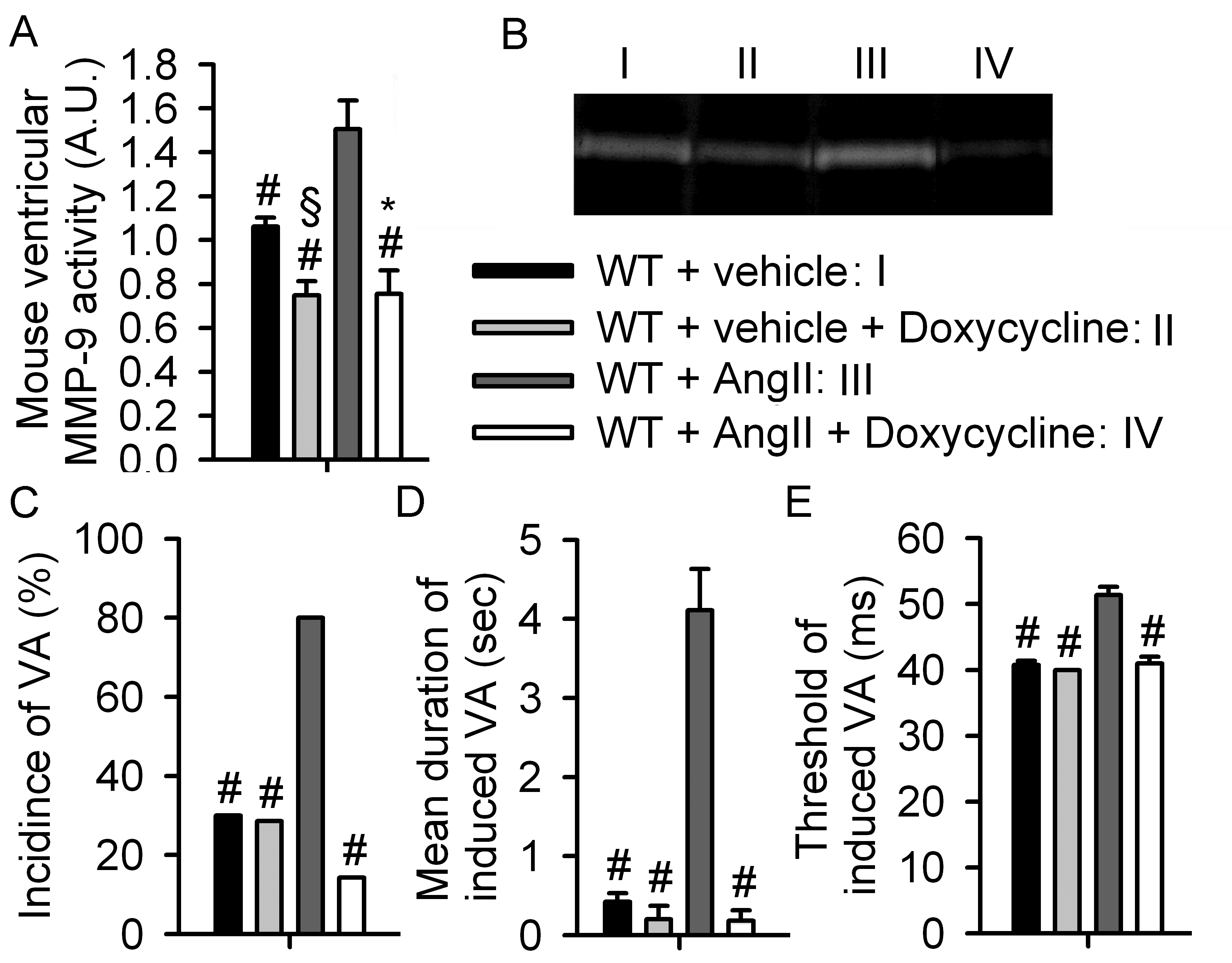
**

(A) MMP-9 enzymatic activity in ventricular tissue, as assessed by zymography (n=5-7, **P*<0.05 vs. WT + vehicle, §*P*=0.052 vs. WT + vehicle, #*P*<0.05 vs. WT + Ang II). (B) Representative image of ventricle MMP-9 activity in different groups. (C to E) Effects of doxycycline treatment. The incidence (C), mean duration (D) and threshold (E) required to induce ventricular arrhythmia were attenuated by feeding the mice doxycycline (n=7-10, #*P*<0.05 vs. WT + Ang II). Data are shown as the mean±SEM.

**Supplementary Figure S10. Full-length blots for Figure 1B.**

**
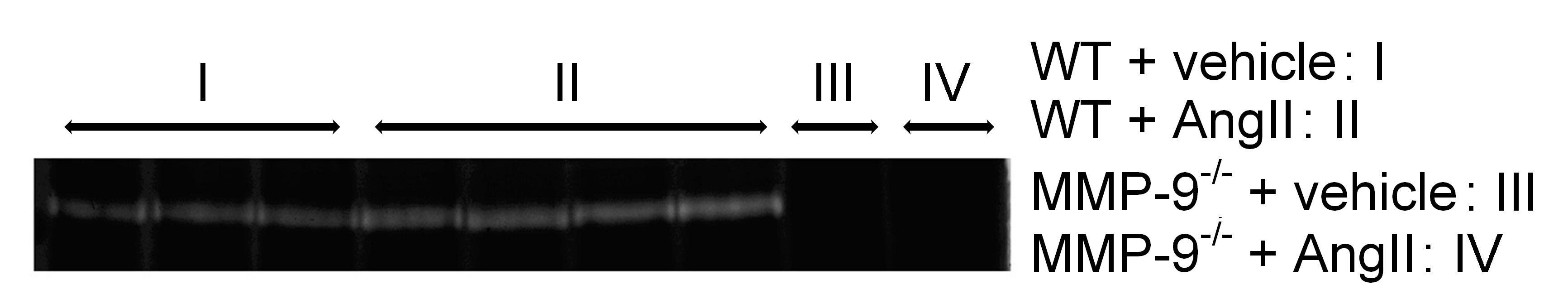
**

**Supplementary Figure S11. Full-length blots for Figure 2G.**


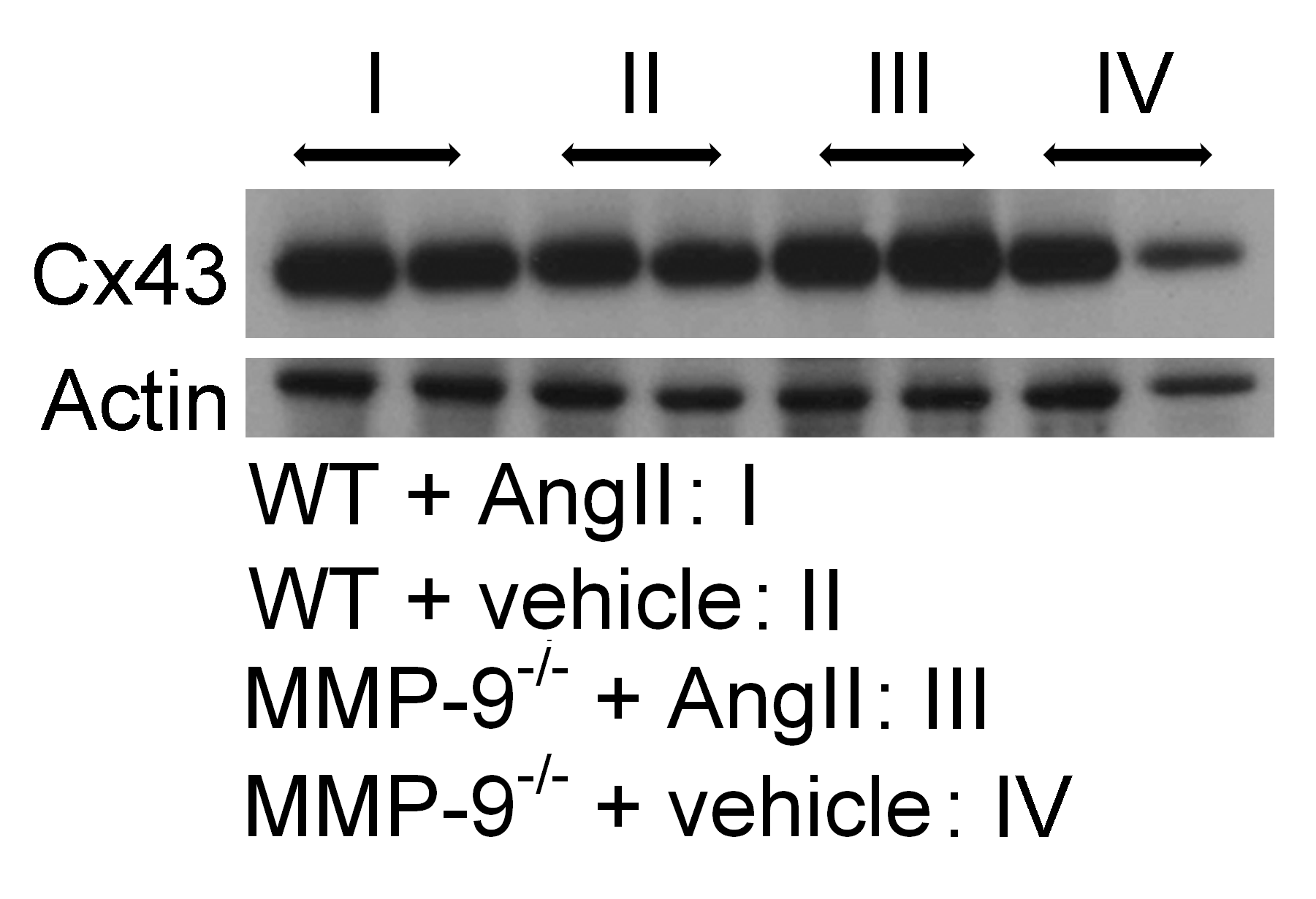


**Supplementary Figure S12. Full-length zymography for Supplementary Figure S9B.**


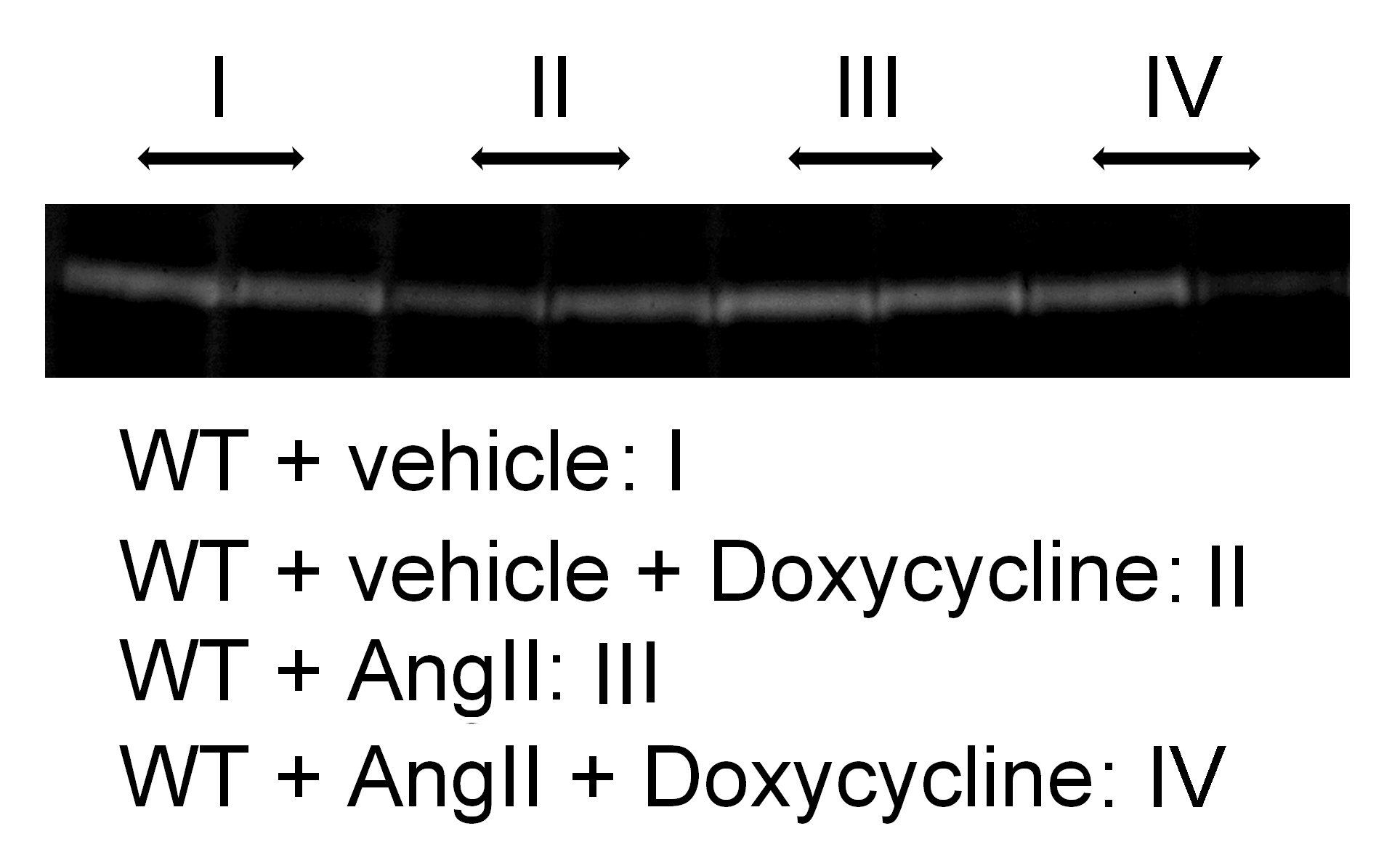


**Supplementary references**

1. Fukui, A. et al. Role of leptin signaling in the pathogenesis of angiotensin II-mediated atrial fibrosis and fibrillation. *Circ Arrhythm Electrophysiol* **6**, 402-9 (2013).

2. Shi, Y. et al. Regulatory T cells protect mice against coxsackievirus-induced myocarditis through the transforming growth factor beta-coxsackie-adenovirus receptor pathway. *Circulation* **121**, 2624-34 (2010).

3. Schluter, K.D. & Schreiber, D. Adult ventricular cardiomyocytes: isolation and culture. *Methods Mol Biol* **290**, 305-14 (2005).

4. Spencer, C.I. et al. Calcium transients closely reflect prolonged action potentials in iPSC models of inherited cardiac arrhythmia. *Stem Cell Reports* **3**, 269-81 (2014).

5. Tsai, W.C. et al. Small conductance calcium-activated potassium current and the mechanism of atrial arrhythmia in mice with dysfunctional melanocyte-like cells. *Heart Rhythm* **13**, 1527-35 (2016).

6. Wu, K.D., Bungard, D. & Lytton, J. Regulation of SERCA Ca2+ pump expression by cytoplasmic Ca2+ in vascular smooth muscle cells. *Am J Physiol Cell Physiol* **280**, C843-51 (2001).

7. Wray, S. & Burdyga, T. Sarcoplasmic reticulum function in smooth muscle. *Physiol Rev* **90**, 113-78 (2010).

8. Liang, H. et al. Regulation of angiotensin II-induced phosphorylation of STAT3 in vascular smooth muscle cells. *J Biol Chem* **274**, 19846-51 (1999).
